# Supplementary material for: Non-avian theropod phalanges from the marine Fox Hills Formation (Maastrichtian), western South Dakota, USA
Source: PeerJ. 2023 Feb 7;11:e14665. doi: 10.7717/peerj.14665 (PMC9912944; doi:10.7717/peerj.14665)
Supplement: Supplemental Information 1 [file peerj-11-14665-s001.docx]

The raw data for this paper consists of two theropod phalanges: one from the Fairpoint Member of the Fox Hills Formation in White Owl, Meade County, South Dakota; the other from the Iron Lightning Member of the Fox Hills Formation near Iron Lightning, Ziebach County, South Dakota. The Fairpoint specimen from White Owl, Meade County, South Dakota has been deposited in the vertebrate paleontology collections of the Denver Museum of Nature and Science (DMNH), Denver, Colorado, USA, and is identified by the catalogue number: DMNH EPV.138575. The Iron Lightning specimen from Iron Lightning in Ziebach County, South Dakota, is in the vertebrate paleontology collections of the Yale Peabody Museum (YPM), Yale University, New Haven, Connecticut, USA, and carries the catalogue number: YPM VP.061705.

Physical attributes of these specimens are described in Table 1.
